# Quality of life in patients with stable coronary artery disease submitted to percutaneous, surgical, and medical therapies: a cohort study

**DOI:** 10.1186/s12955-021-01886-7

**Published:** 2021-11-24

**Authors:** Lucas Molinari Veloso da Silveira, Adriana Silveira Almeida, Felipe C. Fuchs, Aline Gonçalves Silva, Marcelo Balbinot Lucca, Samuel Scopel, Sandra C. Fuchs, Flávio D. Fuchs

**Affiliations:** 1 Postgraduate Studies Program in Cardiology, School of Medicine, Hospital de Clinicas de Porto Alegre, Universidade Federal do Rio Grande do Sul (UFRGS), RS Porto Alegre, Brazil; 2 Division of Cardiovascular Surgery, Instituto do Coração do Hospital das Clínicas da Faculdade de Medicina da Universidade de São Paulo (InCor-HCFMUSP), SP São Paulo, Brazil; 3 Division of Cardiology, Hospital de Clinicas de Porto Alegre, Porto Alegre, RS Brazil; 4 Hospital de Clínicas de Porto Alegre, INCT PREVER, CPC, 5º. and., Ramiro Barcelos, Porto Alegre, RS 2350, 90035-903 Brazil

**Keywords:** Cardiac surgery, Percutaneous coronary intervention, Stable coronary artery disease, Quality of life, Cohort study

## Abstract

**Background:**

Clinical, surgical, and percutaneous strategies similarly prevent major cardiovascular events in patients with stable coronary artery disease (CAD). The possibility that these strategies have differential effects on health-related quality of life (HRQoL) has been debated, particularly in patients treated outside clinical trials.

**Methods:**

We assigned 454 patients diagnosed with CAD during an elective diagnostic coronary angiography to coronary artery bypass grafting (CABG), percutaneous coronary intervention (PCI), or optimal medical treatment (OMT), and followed them for an average of 5.2 ± 1.5 years. HRQoL was assessed using a validated Brazilian version of the 12-Item Short-Form Health Survey questionnaire. The association between therapeutic strategies and quality of life scores was tested using variance analysis and adjusted for confounders in a general linear model.

**Results:**

There were no differences in the mental component summary scores in the follow-up evaluation by therapeutic strategies: 51.4, 53.7, and 52.3 for OMT, PCI, and CABG, respectively. Physical component summary scores were higher in the PCI group than the CABG and OMT groups (46.4 vs. 42.9 and 43.8, respectively); however, these differences were no longer different after adjustment for confounding variables.

**Conclusion:**

In a long-term follow-up of patients with stable CAD, HRQoL did not differ in patients treated by medical, percutaneous, or surgical treatments.

## Background

Coronary artery disease (CAD) is expected to persist as a primary cause of death worldwide until at least 2030 [1]. Clinical, surgical, and percutaneous strategies have demonstrated effectiveness in relieving clinical manifestations and preventing recurrence and fatalities, particularly in acute events [2]. The identification of the best therapeutic strategy in patients with stable CAD, however, remains controversial.

Randomized controlled trials [3, 4] and their meta-analyses [5, 6] demonstrated no evidence of the superiority of interventional treatments over clinical treatment to prevent major cardiovascular (CV) events in patients with stable CAD. The ISCHEMIA and ISCHEMIA-CKD Trials reported that in patients with stable CAD and moderate or severe ischemia, an invasive strategy (coronary artery bypass grafting [CABG] or percutaneous coronary intervention [PCI]), compared with a conservative strategy (optimal medical therapy [OMT] only) did not reduce the incidence of CV events or death after a follow-up of approximately 3 years [7, 8].

However, most clinical trials evaluating strategies for management of chronic CAD compared patients submitted to CABG and percutaneous coronary intervention (PCI), but not medical treatment. Overall, these trials demonstrated no significant difference in mortality or myocardial infarction incidence in patients treated with either of the invasive options. However, surgical patients had lower rates of new revascularization procedures during follow-up [9–11].

On the other hand, observational studies comparing CABG with PCI suggested that the former can be more effective in preventing major CV events [12–14]. However, in studies that included patients treated clinically, the incidence of CV events was not substantially different from patients treated by CABG or PCI, as we demonstrated in a cohort study [15].

Independent of the effectiveness of therapies to prevent major CV outcomes, it is critical to determine whether patients treated with different strategies have better outcomes concerning the frequency of symptoms and quality of life (QoL). The COURAGE Trial [3] and the ISCHEMIA Trial [16] addressed these issues. These trials demonstrated a better health-related quality of life (HRQoL) in patients treated with invasive strategies than conservative strategies with OMT only. In comparing the effects of invasive strategies, CABG may have a more durable benefit over HRQoL than PCI, as demonstrated in a sub-analysis of the SYNTAX Trial [17].

Observational studies with all comers could offer insights regarding the effect of therapies on HRQoL. There are a few observational studies comparing the effect of various treatment strategies in participants subjected to OMT, CABG, or PCI [18–21]. These studies had small sample sizes, high rates of loss to follow-up [18, 19], short follow-up [18, 21], used different scales for the assessment of HRQoL and were not adjusted for confounders [19–21]. A meta-analysis identified these and 30 other observational studies assessing the effect of different treatments over HRQoL [22]. Six studies compared HRQoL in patients treated with PCI or CABG [18–21, 23, 24], and the others reported the effect of individual therapies over HRQoL (after treatment of all participants). A systematic review did not precede this meta-analysis [22], and compared findings of different study arms without the methods recommended for network meta-analysis, separating arbitrarily for analysis studies with and without outliers. Herein, we report a comparison of QoL measurements in a cohort of patients with stable CAD treated with medical, percutaneous, or surgical strategies.

## Methods

Details of the study protocol were described previously [15]. In summary, patients were referred by cardiologists and clinicians for elective diagnostic coronary angiography to a university-affiliated tertiary referral hospital from 2006 to 2014. All patients with a documented diagnosis of CAD were included irrespective of the type of treatment (OMT, PCI, or CABG). The SYNTAX score (SXscore) was calculated prospectively in all patients. Two interventional cardiologists, blinded to clinical characteristics and trained according to the SXscore tutorial, performed the visual angiographic analysis and calculated the scores. In case of disagreement, a third interventionist was consulted, and the final decision was reached by consensus. The option of the therapeutic strategy was chosen by the attending physician and, in more complex cases, after a discussion with a CV surgeon and an interventional cardiologist. We excluded patients with acute coronary syndromes, valvular heart disease, aortic diseases, previous coronary revascularization, class III or IV heart failure, chronic renal disease (previous medical diagnosis or serum creatinine greater than 1.5 mg/dL), history of cancer, or severe psychiatric illness.

A standardized questionnaire was provided immediately before the coronary angiography. This was considered the baseline interview, and it evaluated demographic information, educational history, lifestyle characteristics, and past medical history.

The follow-up of patients was performed through telephone interviews, medical records review, death certificates, and next-of-kin interviews. A combination of strategies was adopted to minimize losses, including contacting patients by registered letters and interviewing the attending physicians.

The outcome in this analysis was HRQoL, assessed using a validated Brazilian version of the 12-Item Short-Form Health Survey (SF-12) questionnaire [25], which uses 12 questions to assess the influence of eight health domains to score physical and mental health dimensions in the four weeks before the interview. The physical health-related domain investigated general health, physical functioning, physical role, and body pain. The mental health-related scales included vitality, social functioning, emotional role, and mental health. We also calculated physical component summary (PCS) and mental component summary (MCS) scores [26–28].

All data were evaluated by at least two authors independently, with quality control on data entry, and checking amplitude and consistency of the variables. For quality control of the team’s performance, 20% of the protocols were randomly selected to be reviewed by a senior investigator (SCF). The reporting was based on Strengthening the Reporting of Observational Studies in Epidemiology guidelines [29].

### Statistical analysis

Results are expressed as mean ± standard deviation and number (percentage) for continuous and categorical variables, respectively. As appropriate, continuous and dichotomous variables were analyzed using the Student’s t test, one-way analysis of variance (ANOVA), or chi-square test. When necessary, the Bonferroni test was applied to identify differences in multiple comparisons. The association between therapeutic strategies and scores of HRQoL were tested by ANOVA and adjusted for confounding using a general linear model. Variables included in the model were theoretically associated with worse clinical outcomes in patients with coronary heart disease and, therefore, with the potential to confound the association of the interventions with scores of HRQoL. Statistical analyses were performed using SPSS, Version 18.0 (SPSS, Inc., Chicago, IL, USA).

### Ethical aspects

The hospital's ethics committee approved the study protocol. The Office for Human Research Protections accredited the committee as an institutional review board, registered under no. 13–0171. All participants provided informed written consent.

## Results

**Fig. 1:**
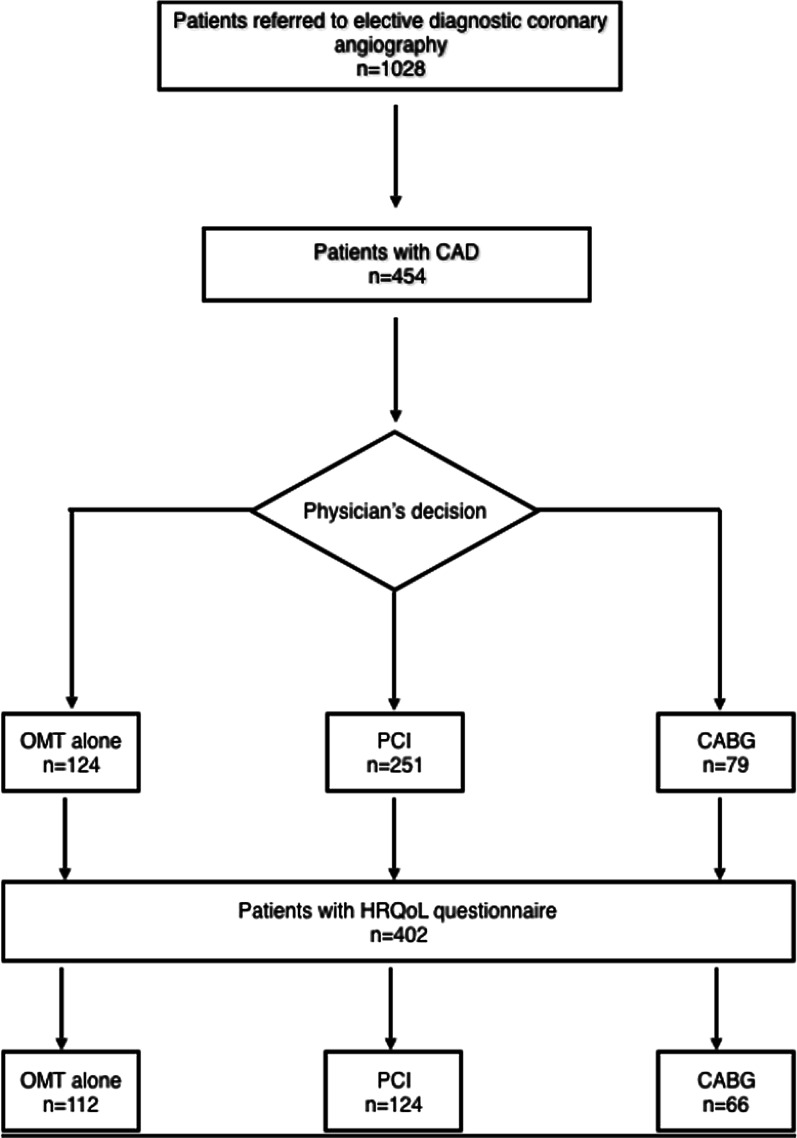
Study flow chart

Among 1028 patients referred for elective diagnostic coronary angiography at our institution during the study period, 454 had a confirmed diagnosis of CAD and were treated by clinical, surgical, or percutaneous interventions. A total of 402 patients (88.5%) completed the HRQoL questionnaire with an average follow-up of 5.2 ± 1.5 years and were included in the analysis. Of these, 112 received OMT alone, 224 underwent PCI, and 66 underwent CABG; (Fig. 1). Participants were an average of 60.8 years old, and most were men (n = 258; 64%). The follow-up time by treatment was 5.1 ± 1.4 years for patients treated clinically (ranging 2.5–8.3 years), 5.3 ± 1.5 (ranging 2.5–8.4) years for patients treated with PCI, and 4.7 ± 1.4 (ranging 2.4–7.4) years for patients treated with CABG.

Table 1 shows that the characteristics of the study population were relatively similar across treatment groups. Differences that should be highlighted were the higher prevalence of women in the OMT group than the PCI and CABG groups (46.4% vs. 31.2% and 33.3%, respectively). Participants who reported a previous myocardial infarction more often underwent interventional treatment (60.6% and 48.7% vs. 31.3%, for CABG, PCI, and OMT alone, respectively). Patients treated with CABG had higher SXscores than the PCI and OMT alone groups (20.5, 9.3, and 6.6, respectively).

**Table.1:** Baseline clinical and angiographic characteristics

Baseline characteristics	MT alone (n = 112)	PCI (n = 224)	CABG (n = 66)	P value
Age (years)	61.2 ± 10.0	60.6 ± 9.1	61.3 ± 8.1	0.79
Male	60 (53.6%)	154 (68.8%)	44 (66.7%)	0.02
Race white	77 (68.8%)	156 (69.6%)	54 (81.8%)	0.13
Years at school (years)	6.1 ± 4.0	6.9 ± 4.3	6.8 ± 4.1	0.21
BMI (kg/m^2^)	29.2 ± 4.8	28.1 ± 4.3	27.8 ± 4.2	0.07
SBP (mmHg)	140.4 ± 22.5	141.6 ± 24.1	141.9 ± 19.8	0.88
DBP (mmHg)	79.3 ± 11. 7	81.6 ± 13.1	81.6 ± 11.6	0.26
Glucose (mg/dL)	103.5 ± 32.4	105.7 ± 27.2	116.3 ± 49.3	0.03
HDL-C (mg/dL)	41.8 ± 10.6	39.7 ± 10.1	40.6 ± 11.4	0.24
Triglycerides (mg/dL)	140.2 ± 81.4	148.8 ± 124.3	156.9 ± 130.6	0.63
Diabetes melliitus	36 (32.1%)	60 (26.8%)	26 (39.4%)	0.13
Hypertension	103 (92.0%)	213 (95.1%)	63 (95.5%)	0.46
Previous myocardial infarction	35 (31.3%)	109 (48.7%)	40 (60.6%)	< 0.001
HF	14 (12.5%)	30 (13.4%)	14 (21.2%)	0.22
Creatinine (mg/dL)	0.68 ± 0.18	0.69 ± 0.18	0.72 ± 0.22	0.44
Current smoking	13 (11.6%)	30 (13.4%)	3 (4.5%)	0.14
SXscore	6.6 ± 8.6	9.3 ± 6.9	20.5 ± 9.7	< 0.001

^*^Variables were described as mean ± SD or number (%)

**Fig. 2:**
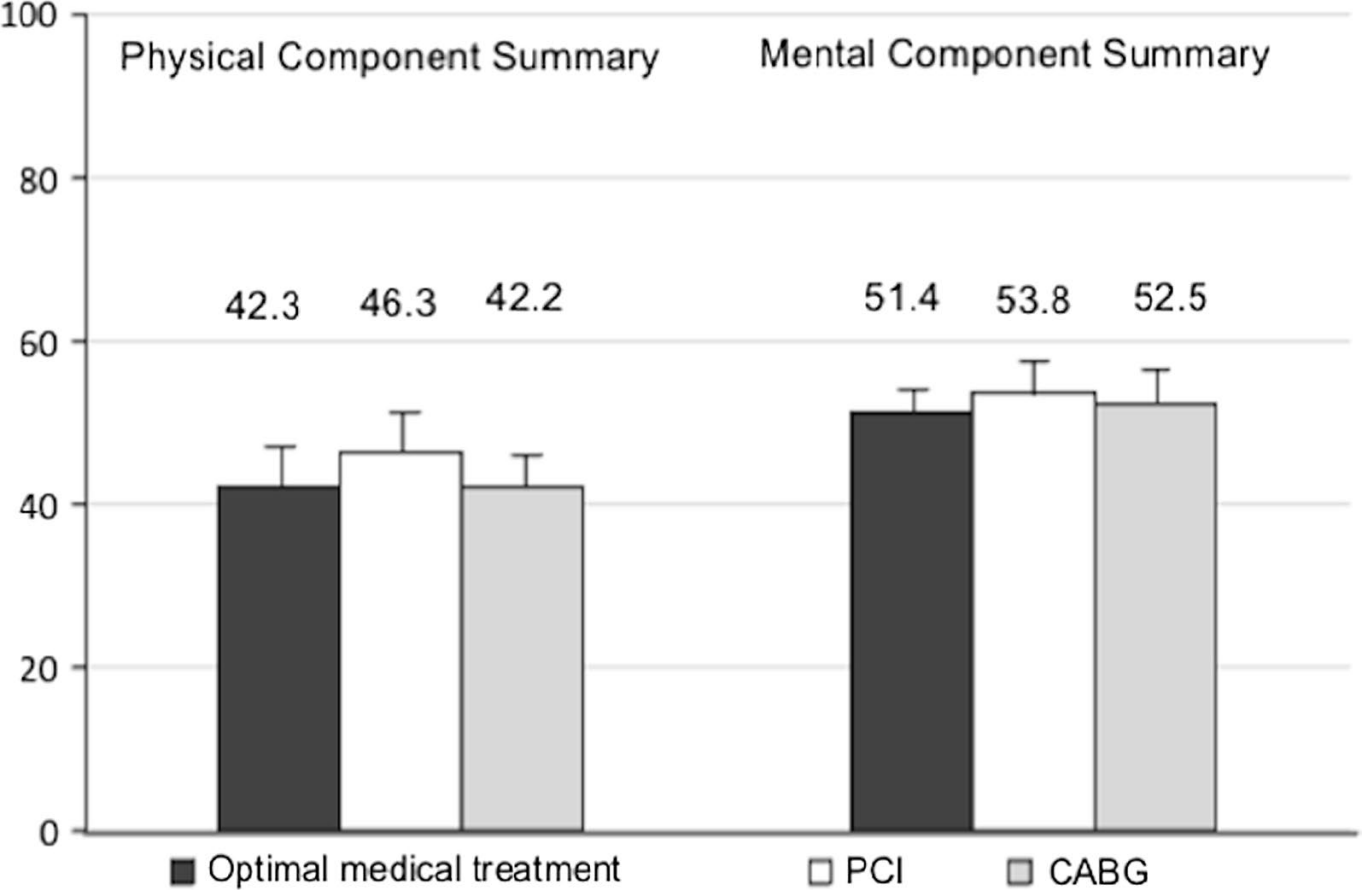
Average SF-12 for mental and physical component summaries, SF_12 score according to treatment

The unadjusted mean values of PCS and MCS scores according to treatment strategy are shown in Table 2. There was no difference in MCS among the three groups, with mean MCS for OMT alone, PCI, and CABG of 51.4, 53.7, and 52.3, respectively. PCS scores in patients treated by OMT alone, PCI, and CABG were shown in Fig. 2, and the score was significantly higher in the PCI group than the CABG or OMT groups. The statistically significant differences shown in Table 2 were no longer significant after adjusting for confounding factors (Table 3).

**Table.2:** Unadjusted mean for quality of life scores after CAD treatment in 402 patients undergoing elective coronary angiography

Treatment	PCS*	MCS
	Mean ± SD (95% CI)	Mean ± SD (95% CI)
OMT alone	43.8 ± 10.5 (41.8–45.8)	51.4 ± 10.8 (49.4–53.4)
PCI	46.4 ± 11.2 (45.0–48.0)	53.7 ± 9.4 (52.5–54.9)
CABG	42.9 ± 11.7 (40.0–45.8)	52.4 ± 10.4 (49.8–54.9)

*PCS* physical component summary score, *MCS* mental component summary score of the SF-12

^*^Between groups ANOVA P value = 0.02

**Table.3:** Mean quality of life scores after CAD treatment in 402 patients undergoing elective coronary angiography after adjustment for confounding

Treatment	PCS*	MCS
	Mean (95%CI)	Mean (95%CI)
OMT alone	44.3 (42.1–46.4)	51.4 (49.5–53.3)
PCI	46.2 (44.8–47.7)	53.8 (52.4–55.1)
CABG	42.2 (39.1–45.2)	52.4 (49.8–55.2)

*PCS* physical component summary score, *MCS* mental component summary score of the SF-12

^*^The differences between groups were not significant after adjustment for age, mean SBP, Syntax score, years at school and BMI

## Discussion

In this cohort study of patients with stable CAD treated clinically or by invasive strategies, we found that all treatment options had similar effects on HRQoL after an average follow-up of 5.2 ± 1.5 years. The trend toward better HRQoL in patients treated through PCI was no longer significant after adjustment for the baseline severity of disease and other confounders.

HRQoL is a multidimensional concept that is subjectively perceived and embraces physical, social, emotional, and functional health [30]. Traditional outcomes of randomized controlled trials and cohort studies may not capture the impact of the intervention on HRQoL. Therefore, HRQoL assessment has become increasingly important in managing patients with CAD, a chronic disease that classically impairs functional capacity and HRQoL [19, 31]. Patients often consider the quality of the additional life-years gained as important as the length of life [32].

The COURAGE Trial was one of the first studies conducted in patients with stable CAD that evaluated HRQoL changes according to treatment strategies (PCI *vs*. OMT alone). In that trial, the initial improvement in HRQoL in patients treated with PCI was no longer detected after 12 months [3]. The benefit of invasive strategies over OMT in terms of HRQoL was reported in another study [33]. In another report, patients undergoing revascularization by CABG had more prolonged improvement in HRQoL than patients treated with PCI [34]. The effect of treatments over angina-related health status, assessed using the Seattle Angina Questionnaire (SAQ) and HRQoL, assessed by European Quality of Life–5 Dimensions in ISCHEMIA Trial, was an a priori sub-analysis specified by the protocol. Participants treated with CABG or PCI had higher SAQ summary scores than patients treated clinically during a follow-up of 36 months [16]. HRQoL, however, improved similarly during the period [16]. The loss of beneficial effects of invasive approaches over HRQoL with longer follow-up in the COURAGE and ISCHEMIA trials suggest that the short-term effects may be at least in part explained by a placebo effect. Our findings also suggest that any eventual benefit of PCI and CABG at short-term follow-up in the real-world care of patients also vanishes with longer follow-up.

Our findings are hardly comparable to the observational studies that assessed the effect of OMT, PCI, or CABG. In addition to the limitations of a few studies that compared the three strategies in contemporary cohorts [18–21], they included short follow-up. To the best of our knowledge, there is only one recent systematic review with meta-analysis addressing this topic [22]; however, non-adherence to the core methods of meta-analyses threatens its internal validity.

Our study has limitations that deserve mention. We did not assess the HRQoL at baseline, and this fact might introduce bias in the assessment of HRQoL at follow-up. Nonetheless, the underlying reasons for differences in HRQoL at the baseline were controlled in the multivariate analysis. Limited statistical power due to the sample size may have concealed a beta error. The study was carried out in only one center, which may reduce its external validity. Nevertheless, our service's patient characteristics and diagnostic and therapeutic practices do not differ substantially from those of other centers. The strengths of our study are that we studied all comers without limitations for participation in clinical trials, we compared three treatment strategies, and there was a prolonged follow-up.

## Conclusion

The HRQoL of patients with stable CAD does not differ after treatment with CABG, PCI, or OMT alone after a relatively long follow-up period. Considering that these strategies have similar effectiveness in preventing major CV outcomes, the option for OMT alone appears to be adequate as the first option for the management of patients with stable CAD.

## Data Availability

All data relevant for this work are available to the community upon reasonable request to the corresponding author.

## Abbreviations

CABG: Coronary artery bypass grafting
CAD: Coronary artery disease
CV: Cardiovascular
EQ-5D: European Quality of Lifeâ€“5 Dimensions
HRQoL: Health-related quality of life
MCS: Mental Component Summary
OMT: Optimized medical therapy
PCI: Percutaneous coronary intervention
PCS: Physical component summary
SAQ: Seattle Angina Questionnaire
SD: Standard deviation
SF-12: 12-Item Short-Form Health Survey
STROBE: Strengthening the Reporting of Observational Studies in Epidemiology
SXscores: Syntax Scores
